# Inhibition of Miro1 disturbs mitophagy and pancreatic β-cell function interfering insulin release via IRS-Akt-Foxo1 in diabetes

**DOI:** 10.18632/oncotarget.20963

**Published:** 2017-09-16

**Authors:** Lingling Chen, Chunyan Liu, Jianfeng Gao, Zhiwen Xie, Lawrence W.C. Chan, Damien J. Keating, Yibin Yang, Jiazhong Sun, Fuling Zhou, Yongchang Wei, Xiuli Men, Sijun Yang

**Affiliations:** ^1^ ABSL-3 Laboratory at the Center for Animal Experiment and Institute of Animal Model for Human Disease, Wuhan University School of Medicine, Wuhan, P. R. China; ^2^ Department of Cell Biology, College of Life Science, Nanjing Normal University, Nanjing, Jiangsu, P.R. China; ^3^ School of Bioscience and Technology , Weifang Medical University, Weifang Shandong, P.R. China; ^4^ Department of Health Technology and Informatics, Hong Kong Polytechnic University, Hong Kong, Hong Kong; ^5^ Department of Human Physiology and Centre for Neuroscience, Flinders University, Adelaide, South Australia, Australia; ^6^ Department of Endocrinology, Zhongnan Hospital of Wuhan University, Wuhan, Hubei, P.R. China; ^7^ Department of Respiratory, Zhongnan Hospital of Wuhan University, Wuhan, Hubei, P.R. China; ^8^ Department of Hematology and Radiation, Zhongnan Hospital of Wuhan University, Wuhan, Hubei, P.R. China; ^9^ Department of Medical Oncology, Zhongnan Hospital of Wuhan University, Wuhan, Hubei, P.R. China; ^10^ School of Basic Medical Sciences, North China University of Science and Technology, Tangshan, P.R. China

**Keywords:** Miro1, mitophagy, insulin resistance, HFD, diabetes, Pathology Section

## Abstract

Mitochondrial function is essential to meet metabolic demand of pancreatic beta cells respond to high nutrient stress. Mitophagy is an essential component to normal pancreatic β-cell function and has been associated with β-cell failure in Type 2 diabetes (T2D). Our previous studies have indicated that mitochondrial Rho (Miro) GTPase-mediated mitochondrial dysfunction under high nutrient stress leads to NOD-like receptor 3 (NLRP3)-dependent proinflammatory responses and subsequent insulin resistance. However, the *in vivo* mechanism by which Miro1 underlies mitophagy has not been identified. Here we show firstly that the expression of Miro is reduced in human T2D and mouse db/db islets and in INS-1 cell line exposed to high glucose and palmitate. β-cell specific ablation of Miro1 (Miro1f/f: Rip-cre mice, or (IKO) under high nutrient stress promotes the development of hyperglycemia. β-cells from IKO mice display an inhibition of mitophagy under oxidative stress and induces mitochondrial dysfunction. Dysfunctional mitophagy in IKO mice is represented by damaged islet beta cell mitochondrial and secretory capacity, unbalanced downstream MKK-JNK signalling without affecting the levels of MEK, ERK or p38 activation and subsequently, impaired insulin secretion signaling *via* inhibition IRS-AKT-Foxo1 pathway, leading to worsening glucose tolerance in these mice. Thus, these data suggest that Miro1 may be responsible for mitophagy deficiency and β-cell dysfunction in T2D and that strategies target Miro1 *in vivo* may provide a therapeutic target to enhance β-cell mitochondrial quality and insulin secretion to ameliorate complications associated with T2D.

## INTRODUCTION

Excessive caloric or fat intake leading to obesity has been associated with beta cell dysfunction, and subsequently insulin resistance and T2D (type 2 diabetes) in human . High fat diet (HFD) feeding in mice has been reported to cause deficient islet beta cell mitophagy [1, 2]. Mitochondrial function regulated by mitophagy is essential to preserve pancreatic beta cell function and insulin secretion. Impaired beta cell function, together with deficient mitophagy, are both associated with chronic inflammation and insulin resistance [3]. Thus, insulin resistance, inflammation and pancreatic cell dysfunction are common pathological events in obese individuals [4]. Although in-depth research has been done in this area, the occurrence of complex and associated cell events and related behaviors *in vivo* connecting mitochondrial function and perturbed insulin secretion in T2D are not well understood. Mitochondrial activity is necessary for the process of cell proliferation and metabolism, and mitochondrial dysfunction is associated with a variety of human diseases, including cancer [5], diabetes [3] and age-related diseases [6]. Increasing evidence suggests that mitophagy maintains mitochondrial integrity and quality control not only by selectively removing dysfunctional or damaged mitochondria [7-9], but also by promoting biosynthesis of new mitochondria [10]. Recently, it has been found that before the onset of mitophagy, Cells block mitochondrial motility by causing mitochondrial Rho GTPase (Miro) degradation and ubiquitination of mitochondrial proteins to promote their identification and recruitment to autophagosomes [11-14]. Miro1 is a Rho GTPase with a Ca^2+^ binding site [15]. Oxidative stress-induced increases in cellular Ca^2+^ concentrations, a primary target for insulin release; target Miro1 for stop mitochondrial motility. A deficiency in Miro1-mediated mitophagy triggers triggers mtROS(mitochondrial ROS)-induced NLRP3 dependent pro-inflammatory responses and is linked to T2D diseases *in vitro* [12, 15] [9, 16, 17] .

Here, we provide *in vivo* evidence that Miro1 ablation in islets (IKO) results in systemic inflammation in plasma, leading to pronounced hyperglycemia. Islet beta cells from IKO mice under HFD have impairment of insulin secretion. In addition, the ablation of Miro1 in diabetic mice inhibited mitophagy, caused mitochondrial dysfunction, and unbalanced activation of downstream MKK-JNK pathway without affecting the levels of MEK, ERK or p38 resulted in insulin resistance *via* IRS-AKT-Foxo1 inhibition. Thus, ablation of Miro1 in IKO mice interferes with autophagy of islet beta cells and causes mitochondrial dysfuncion and subsequent insulin secretion impairment. This study provides a mechanism for unraveling the role of Miro1 in the pathogenesis of T2D under HFD stress.

## RESULTS

### Miro1 is decreased in pancreatic beta cells and islets under HFD stress

**Figure 1:**
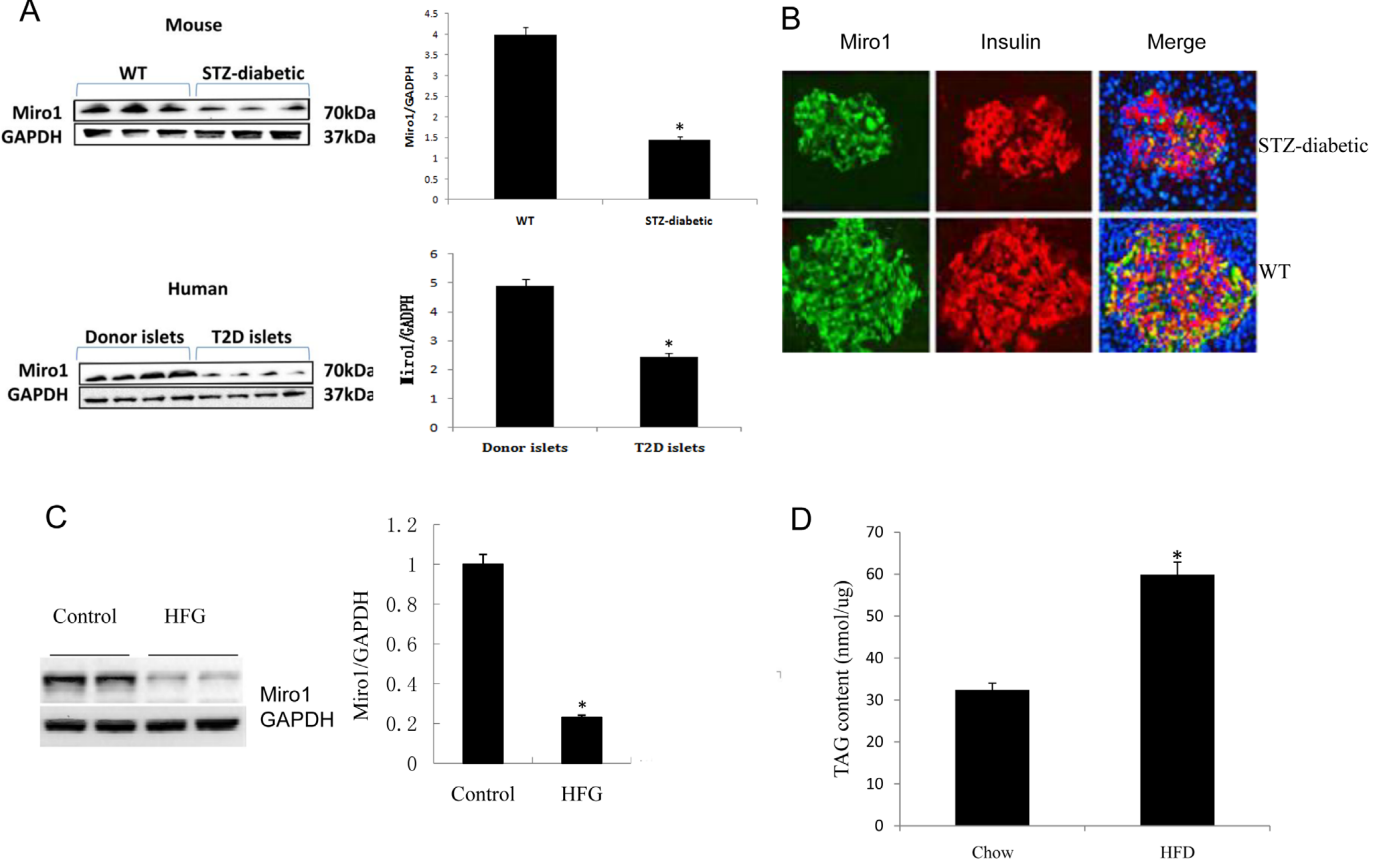
**A.** The protein level of Miro1 in islet samples from diabetic mouse model and biopsy patients with T2D. Miro1 expression was normalized to that of GAPDH (*n* = 4 for normal and 7 for T2D samples, *P < 0.05 *versus* control). **B.** The distribution of the Miro1 within islet cells from STZ-diabetic mouse model was analyzed using confocal microscopy. **C.** Miro1 protein expression in the islet of mice subjected to an HFD for 24 weeks and in NC controls (*n* = 5 samples per experimental group, *P < 0.05 compared with the NC group). GAPDH served as a loading control **D.** TAG contents in the islet of mice subjected to an HFD for 24 weeks and in NC controls (*n* = 6 samples per experimental group, *P < 0.05 *versus* control). The data represent as the mean±SD. Statistical analysis was carried out by Student’s two-tailed *t*-test.

Recent studies have shown that HFD can cause pancreatic beta cell dysfunction and impair insulin release in mice (1). To examine the potential involvement of Miro1 in pathophysiological processes associated with β-cell dysfunction, we detected Miro1 expression in islets from human T2D donors and the db/db mouse models of T2D. Miro1 expression was decreased in the mitochondria in db/db mice and T2D patients (Figure 1A). A similar result was observed in INS(rat insulinoma cell)-823/13 cells under diabetic conditions treated with 0.5mM PA(palmitic acid) + 20mM Glucose (Figure 1C). Previous studies showed that treatment with high glucose and palmitate induced Miro1 degradation *via* a Ca2+-dependent pathway (7). Similar to these results, a decline in Miro1 staining was observed in STZ mice (Supplementary S3) (Figure 1B). In addition, TAG content in islets was increased by more than 30.5% upon HFD feeding (Figure 1D). These observations suggest that Miro1 may play a role in the pathogenesis of β-cell dysfunction.

### Loss of Miro1 results in obesity and insulin resistance

**Figure 2:**
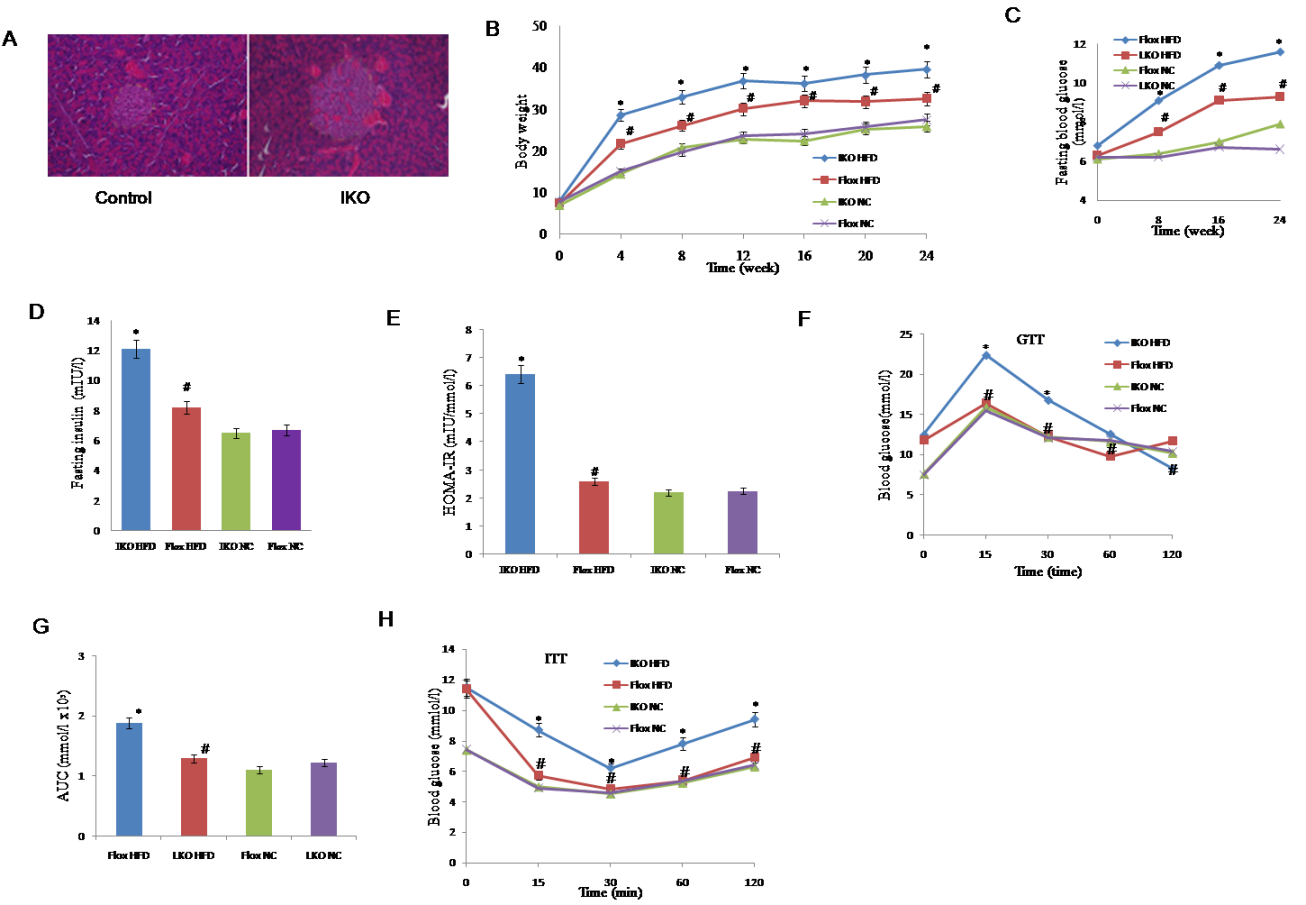
**A.** Haematoxylin and eosin staining of islet sections of IKO and floxed controls. **B.** Changes in the body weight of IKO and floxed mice treated with an HFD or NC for 24 weeks (*n* = 13-18 for each group per time point). **C.**-**E.** FBG, FINS and HOMA-IR in IKO or floxed mice treated with an HFD or NC for 24 weeks. FBG levels were measured every 4 week **C.** FINS was measured at the end point of this experiment **D.** HOMA-IR was calculated as HOMA-IR = (FBG (mmol l−1) × FINS (mIU l−1))/22.5 (*n* = 7-13 for each group per time point)**E.** **F.** Intraperitoneal glucose tolerance test (GTT) (1 g kg−1). **G.**The corresponding area under the curve (AUC) of blood glucose level was calculated (*n* = 7-9 for each group). **H.** Intraperitoneal insulin tolerance test (ITT; 0.75 units per kg) was performed on floxed and IKO mice at the 22nd or 23rd week of food administered, respectively. For A-H, *P < 0.05 compared with the corresponding floxed/NC group; #*P* < 0.05 compared with the corresponding floxed/HFD group. All values are means±SD. Significance determined by two-way analysis of variance with general linear model procedures using a univariate approach **A.**-**D.**, **G.** and Student’s two-tailed *t*-test **E.**, **H.**

Given that the STZ-diabetic mice have decreased level of Miro1 expression in islets, we created Miro1 ablation mice in the islets (IKO) (Supplementary Figure 1) and subjected them to a diabetic condition. Hematoxylin and eosin (H&E) staining and quantification showed that the average islet size of IKO pancreas was larger approximately by 114.5% compared to floxed pancreas (Figure 2A, left and right). In response to HFD treatment, IKO mice significantly increased body weight during 4 to 24 weeks compared to mouse-specific Miro1flox / flox (Floxed) mice (Figure 2B). However, there was no significant difference in the level of energy intake between floxed and IKO mice (Supplementary Figure 2), thus eliminating the lack of Miro1-deficient as a cause of lower body weight in mice. With regard to the effect of islets Miro1 on insulin resistance, We observed fasting blood glucose (FBG) (Figure 2C), fasting serum insulin (FINS) levels (Figure 2D-2E) and homeostasis model assessment of the insulin resistance index (HOMA-IR) (Figure 2F). After HFD treatment, these levels were significantly higher in IKO mice than those in the floxed group. In addition, compared with the NC group, in intraperitoneal glucose tolerance test and intraperitoneal insulin tolerance test assays, the area under the curve was increased in IKO mice, and these effects in floxed significantly improved (Figure 2G-2H). Taken together, we find that reduction Miro1 results in obesity and disturbs insulin release in mice.

### Loss of Miro1 inhibits mitophagy under HFD stress

**Figure 3:**
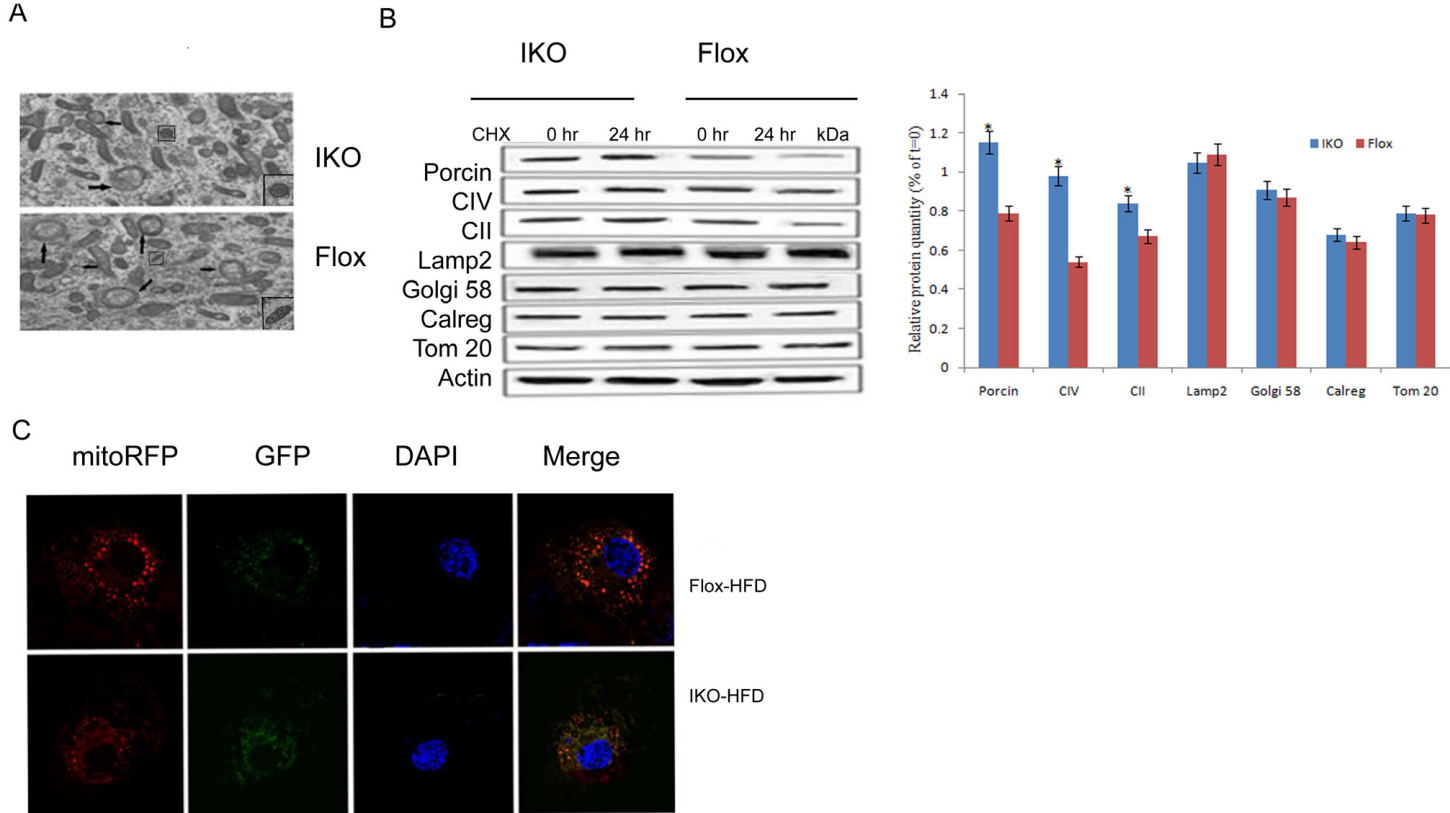
**A.** Electron microscopic analysis of mitochondria showed mitochondrial fragmentation in IKO mice compared with floxed mice. **B.** Protein degradation rates were measured in islet cells from IKO and floxed mice at 0 and 24 h of treatment with 10 mg/ml cycloheximide (CHX). Level of the mitochondrial proteins was analyzed using antibodies against porin, SDHA subunit of complex II (CII), ATP synthase (CV), and Tom20. Golgi apparatus, lysosomes, and endoplasmic reticulum were analyzed using Golgi58, Lamp2, and calregulin (calreg) antibodies. Quantification of protein levels after 24 h CHX treatments on islet cells was showed in down panel. Blue bars are IKO, and red bars are floxed. Values are percentage of respective protein amount at *t* = 0 (*n* = 3). Dashed line represents value at *t* = 0 and normalized to 100% for each protein. *p < 0.05, ***p < 0.001. Data are presented as mean ± SD. **C.**The co-localization of mito-RFP with GFP was observed using confocal microscopy. Cell nuclei were stained with DAPI, and fluorescence signals were visualized by confocal immunofluorescence microscopy. Green fluorescence indicates autophagosomes, and red fluorescence indicates mitochondria.

Damaged or dysfunctional mitochondria removed by mitophagy was one mechanism to maintain mitochondrial health, and the accumulation of those abnormal mitochondria in the cell under HFD pressure may be the underlying cause of the pathogenesis of the disease. Conform to this concept, we investigated the effect of Miro1 knockdown on mitophagy *in vivo*. We used transmission electronmicroscopy (TEM) to confirm the double-membrane structure of autophagosomes containing undigested organelles, to observe whether HFD caused mitochondrial and autophagosome damage. As shown in Figure 3A, oval mitochondria in the control group had a complete crista structure. However, after 24 hours of incubation with HFD, mitochondria swelled and had few cristae in islet beta cells. Double membrane vesicles enclosing the mitochondria were also examined. Further, we measured the contribution of Miro1 to mitophagy in islet cells under diabetic conditions composed of 20 mM glucose and 0.5 mM PA incubation. We treated the islet cells with the protein synthesis inhibitor cycloheximide (CHX) to examine the rate of protein degradation. As a result, mitochondrial protein degradation rates were lower in IKO islet cells with 20 mM glucose +0.5mM PA compared to floxed islet cells. Effect of CHX treatment on protein levels of other cellular compartments such as Golgi (Golgi-58), lysosome (Lamp2), endoplasmic reticulum (calregulin), or cytosol (Gapdh, Actin) were not associated with the Miro1 effects (*, *P* < 0.05). Then, to get further evidence, we detect mitophagy by transfecting multi-functional pLVX-GFP-LC3B-IRES-mito-mCherry vector into islet beta cell. Green fluorescence shows autophagosomes, red fluorescence indicating mitochondria. Yellow fluorescence indicates co-localization of autophagosomes and mitochondria. Obviously, this colocalization was reduced in Miro1 konckdown islet beta cells. These results demonstrated that Miro1 ablation contributed to the markedly reduced in mitophagy under diabetic conditions (Figure 3C).

### Loss of Miro1 promotes damaged-mitochondria-produced ROS and inflammatory responses

**Figure 4:**
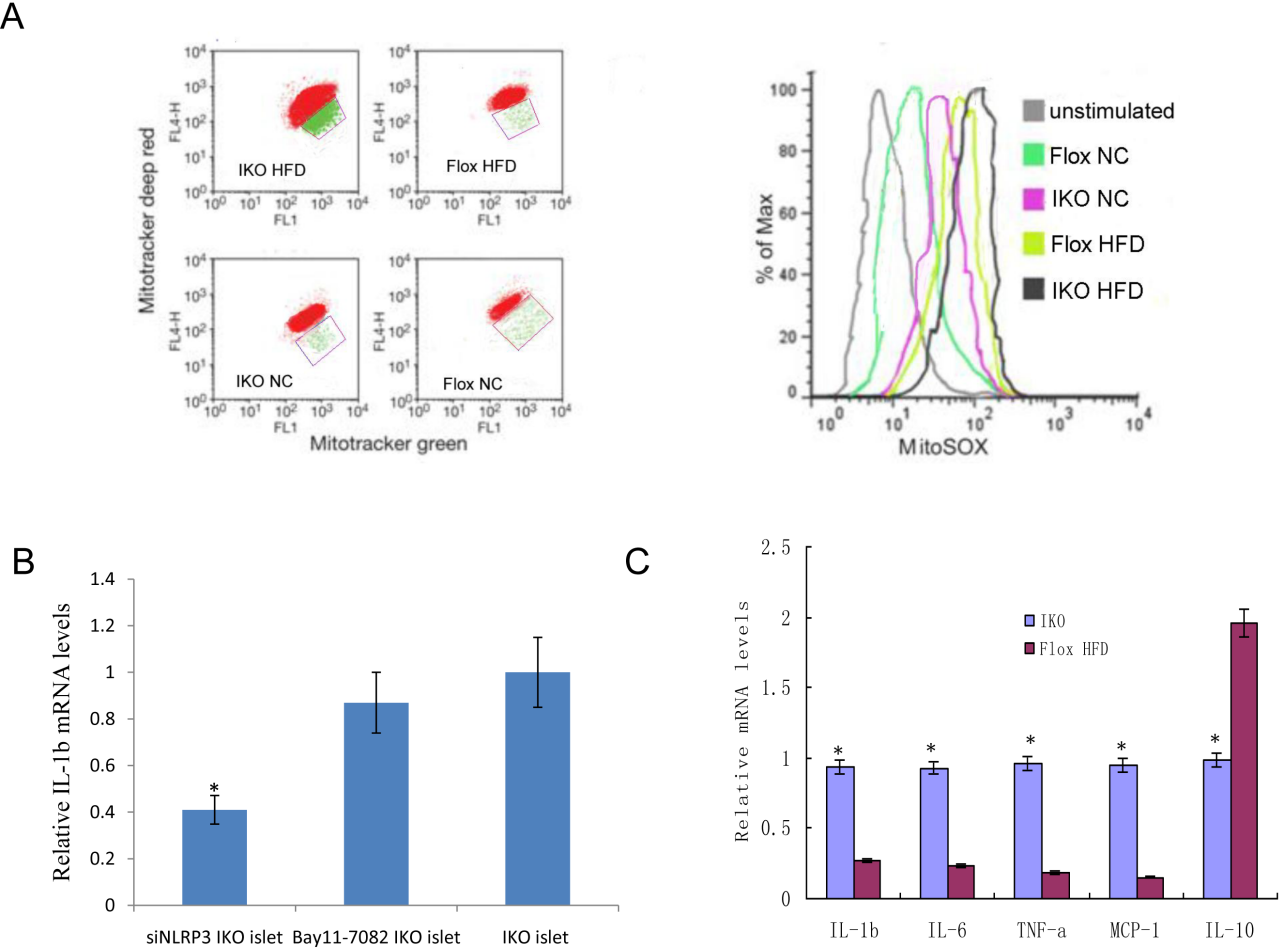
**A.** Islet cells from IKO and floxed mice under HFD/NC treatment were grown for 6 h and then stained with Mitotracker green and Mitotracker deep red or MitoSOX for 30 min and analyzed by flow cytometry. **B.**The IL-1b mRNA expression levels treated by siNLRP3 and Bay11-7082. **C.** Relative mRNA expression levels of the proinflammatory cytokines TNF-α, IL-1β, IL-6, IL-10 and MCP-1 were determined by qRT-PCR in islets from IKO and floxed mice. Mean±SD from 3 independent experiments, analyzed by one-way ANOVA following Newman-Keuls post hoc test. **,*P* < 0.01 compared to the control groups.

To examine whether damaged mitochondrion-producing ROS and proinflammatory responses were induced under HFD stress, we used three types of mitochondria-specific markers to measure the production of mtROS, that distinguished respiring (MitoTracker deep red) which stains strongly depolarized mitochondria, total (MitoTracker green) which stains total weakly polarized mitochondrial membrane potential, and ROS-generating mitochondria (MitoSOX) (Figure 4A, left). As shown in confocal fluorescence microscopy, IKO cells had very higher MitoTracker red staining when compared to floxed cells. Thus, the density of dysfunctional mitochondria, as expressed as MitoTracker red/green ratio, was increased by 43.5% in IKO islet cells (Figure 4A, right). Previous study in our group has identified that the pancreatic islets ROS is mainly produced by mitochondria and NADPH oxidase [15], and the process of producing ROS is a feed-forward vicious cycle. Then, siRNA targeting NLRP3 suppressed IL-1b mRNA expression significantly more than a specific inhibitor of NF-κB (Bay11-7082) targeted NF-KB pathway in HFG(high fat and glucose)-treated IKO islet cells (Figure 4B). These results suggested that NLRP3 not the activation of NF-KB pathway were involved in inflammation activation under HFG diabetic condition. Because T2D is closely related to chronic inflammatory response [7], we measured the expression levels of inflammatory mediators in the serum samples. The serum levels of IL-1b, IL-6, TNF-a and chemokines monocyte chemotactic protein-1 (MCP-1) were significantly higher in IKO mice, but lower in floxed mice after HFD treatment (Figure 4C). Collectively, these findings show that islets from Miro1 KO exacerbate HFD-induced mitochondrial dysfunction and the related inflammatory responses.

### Miro1 ablation damages beta cell mitochondrial and secretory function in mice

**Figure 5:**
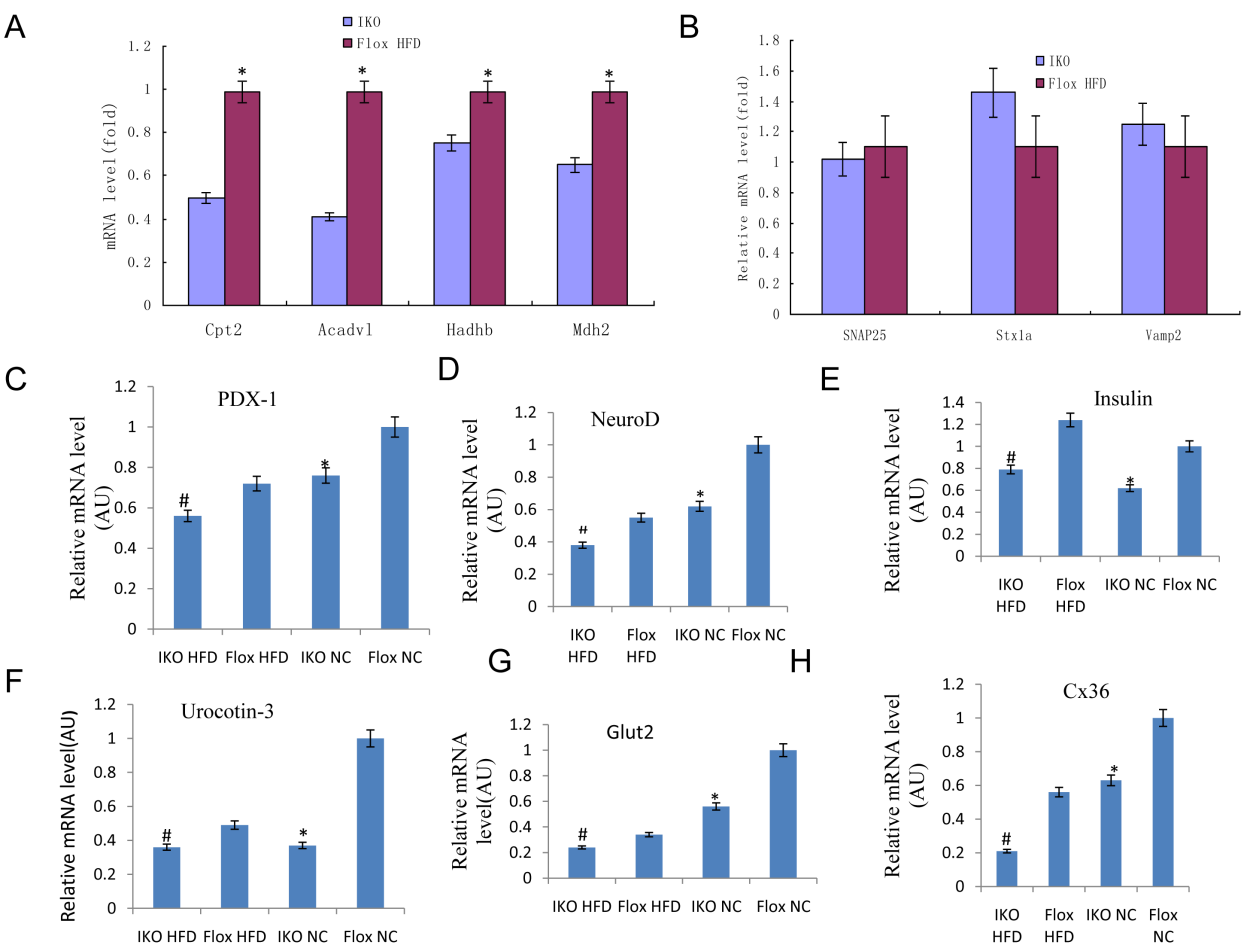
**A.-B.** RT-qPCR of mitochondrial function related genes Cpt2, Acadv1, Hadhb, and Mdh2 A. and unrelated genes SNAP25, Stxla, and Vamp2 B. in isolated islets. **C.**-**H.** mRNA level of genes PDX-1, NeuroD, Insulin, Glut2, Urocortin-3, and Cx36 involved in beta cell function and communication in islets from IKO and Floxed mice either on chow or HFD. mRNA level of each gene was normalized to 18S rRNA level in the same sample. Mean ± SD, *n* = 6 per group. *p < 0.05 flox-NCD *versus* IKO-NCD or flox-NCD *versus* flox-HFD; **p < 0.01 flox-NCD *versus* IKO-NCD or flox-NCD *versus* IKO-HFD; #*p* < 0.05 flox-HFD *versus* IKO-HFD.

Next, we examined expression levels for those genes involved in mitochondrial oxidation, such as Mdh2 and Sdhb, which are involved in the TCA cycle and may regulate GSIS. We examined expression of Cpt2, Acadvl, and Hadhb (Supplementary S3), which are also related to FA oxidation [18]. We found a significant decrease in mRNA levels for all of these genes in IKO islets compared to floxed islets (Figure 5A, top left), while expression of SNAP25, Stx1a, and Vamp2, which are related to exocytosis, was not changed (Figure 5B, top right). Thus, gene expression data also support the notion of the defect in mitochondrial oxidative function in IKO islet cells. To test whether the insulin secretory defect in IKO islet cells is associated with altered gene expression, we performed quantitative RT-PCR analyses. As shown in Figure 5C, the IKO islet cells expressed lower levels of genes involved in normal beta cell secretory function such as PDX-1, NeuroD, insulin, Glut2, and urocortin3(Supplementary S3) [19]. Together, these data suggested that islet beta cell mitochondrial and secretory function was damaged by the ablation of Miro1.

### Unbalanced MKK-JNK signalling is involved in Miro1 impairs insulin signaling *via* IRS-AKT-Foxo1

**Figure 6:**
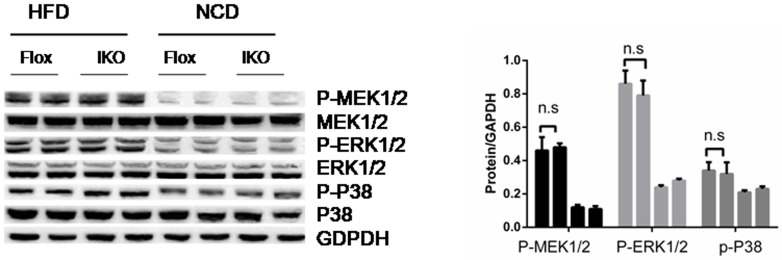
The expression levels of P-MEK1/2, P-ERK, P-p38 and their corresponding total proteins in the islet sample from mice of HFD- or NC-treated mice in Flox and IKO groups for 24 weeks (*n* = 6-7) n.s., no significant difference. All values are means ± s.d. Significance determined by two-way analysis of variance with general linear model procedures using a univariate approach.

**Figure 7:**
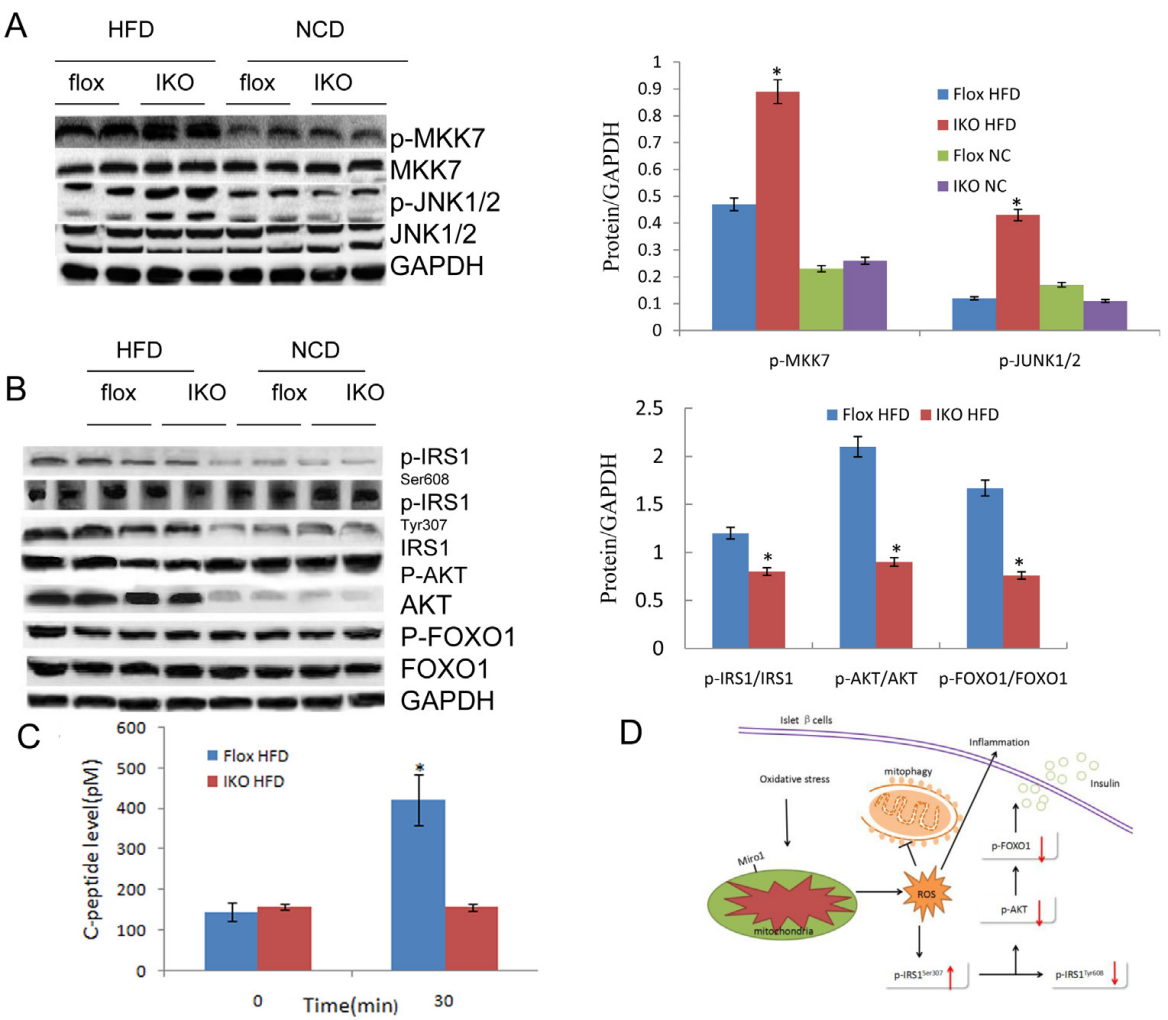
**A.**The expression levels of proteins pathway in JNK signalling in the islet sample from indicated mice. Protein expression was examined by western blotting and normalized to the indicated total protein or GAPDH expression after 4 weeks’ HFD treatment (*n* = 6 samples per experimental group). **B.** Total and/or phosphorylated IRS1, AKT, FOXO1, and GAPDH in islet samples of HFD-fed mice that were refed for 4 h after overnight fasting and NC mice under fasting state at the end of 24 weeks. GAPDH serves as the loading control (*n* = 4 samples per experimental group). All values are means±s.d. Significance determined by two-way analysis of variance with general linear model procedures using a univariate approach. **C.** C-peptide levels in IKO and Flox mice. **D.** Schematic illustration of cellular events underlying Miro1-mediated insulin resistance. In response to a continuous challenge with HFD, Miro1 expression is decreased in islet beta cells leading to the activation of mtROS production and mitophagy inhibition, thereby promoting inflammatory response. The activated JNK further aggravates insulin resistance through enhancing IRS phosphorylation at Ser307 and disrupts its downstream AKT-Foxo1 phosphorylation, which contribute to inflammation and insulin resistance, and the resultant T2D.

The study of the above-mentioned Miro1 involved in the regulation of insulin resistance and related pathology has prompted us to explore its underlying molecular mechanisms. Considering the possible involvement of MAPKs, particularly JNK and p38 MAPK cascades, in which the level of total protein and phosphorylated protein in the signal transduction was measured in the progression of this pathological condition. It was found that phosphorylated MEK, ERK or p38 did not change with the expression of Miro1 (Figure 6) while JNK signaling levels was markedly increased in the islet in response to HFD treatment *in vivo* (Figure 7A). Thus western blotting results showed that ERK, p38 and JNK signalling were activated simultaneously in the islets in response to HFD treatment (Figure 6), whereas the absence of Miro1 only significantly enhanced the activation of the HFD-induced MKK7-JNK-C-JUN pathway induced by *in vivo* (Figure 7A). Previous studies showed that the impairment of insulin sensitivity is primarily mediated by insulin signalling, particularly IRS-AKT-Foxo1. Therefore, we studied the phosphorylation of IRS1, AKT and Foxo1 in these mice that were refed for 4 h after overnight fasting, and observed that IKO mice reduced insulin signal transduction induced by refeeding, as demonstrated by increased phosphorylation of IRS1 at residue Ser307 and decreased phosphorylated levels of IRS1 (Tyr608), AKT, and Foxo1 (Figure 7B) in IKO mouse islets compared to floxed . In general, observation of IKO mice showed that insulin signaling was inhibited due to the specific lack of islets of Miro1. Examination of insulin levels during the first 30 min after glucose administration during the GTT indicated that, unlike IKO groups with a significant increase in insulin levels from 1.3- to 1.5-fold, NC mice were able to raise insulin levels in response to a glucose challenge (Figure 2G-2H). We also measured C-peptide levels, which, due to its longer half-life, can be a more sensitive index for insulin secretion. Indeed, C-peptide levels were elevated by approximately 3-fold 30 min after glucose administration in NC mice, whereas the levels did not change in beta cell IKO mice under HFD (Figure 7C). These results point to the idea that insulin secretory function of beta cells may be defective in IKO mice respond to HFD treatment (Figure 7B). Unaltered glucose tolerance in our RIP-Cre mice probably was due to the pure C57BL/6J background that we employed in our studies. Schematic illustration of molecular events and related behaviors underlying Miro1ablation in islet beta cell is shown in Figure 7C: respond to a continuous HFD stress, Miro1 expression is reduced in beta cells and directly inhibit mitophagy and mitochondrial dysfunction, leading to the activation of downstream MKK7-JNK-C-JUN and inflammatory response. By enhancing the phosphorylation of IRS on Ser307 and destroying its downstream Foxo1 phosphorylation aggravates insulin resistance, which contribute to inflammation and insulin resistance (Figure 6).

## DISCUSSION

In this study, using islet-specific Miro1-KO (IKO) mice, we identified Miro1 in pancreatic beta cell as a negative regulator of HFD-induced mitophagy deficiency, inflammatory responses, leading to beta cell secretory dysfunction. Investigations of the underlying mechanisms have shown that downregulation of the level of expression of Miro1 after a continuous challenge with an HFD, leads to imbalance activation of MKK-JNK-IRS1^307^ downstream and inhibition of AKT-Foxo1 phosphorylation cascades, promotion of IKO mouse pancreatic islet insulin resistance, inflammatory response and metabolic disorders, thereby the resultant T2D (Figure 6).

The mitochondria in beta cells play a key role in producing ROS, regulating intracellular signaling and controlling cell death [20]. Metabolic stress-induced mitochondrial damage and ROS overproduction has been shown to lead to beta cell dysfunction and further develop into T2D [15, 21]. The presence of ROS in HFD stress indicates that mitochondria are the major source of ROS in islet cells after HFD treatment because the effect of DPI (NADPH oxidase inhibitors) on the elimination of HFD-induced ROS production is negligible [22]. It has been demonstrated that mtROS activates NLRP3 inflammasome together with fatty acids *in vitro*, leading to the release of active IL-1β and the production of IL-1-dependent cytokines and chemokines. In our previous study, it was confirmed that Bay11-7082 did not participate in the initiation of NLRP3 inflammatory body, so the NLRP3-dependent proinflammatory response driven by sustained mtROS production was not caused by NF-Kb activation [15] . Consistent with previous reports, in the present study, HFD stress reduced the expression of Islet Miro1, thereby promoting the damage of mitochondrial-induced mtROS production and loss of membrane potential, further leading to cytokines (TNF-α, IL-6 and IL- 1β) and chemokine (MCP-1) in islet β cells of IKO mice [23]. In addition, our findings confirmed that Miro1 ablation damages beta cell function examined by Cpt2, Acadvl, and Hadhb led to inflammasome activation interfering with mitochondrial secretory function by detecting PDX-1, NeuroD, insulin, Glut2, and urocortin3, suggesting a therapeutic promising of Miro1 for mtROS-related T2D.

Mitophagy is an important mechanism for the elimination of damaged mitochondria and the protection of mitochondrial health, and plays a key role in protecting beta cells under metabolic stress conditions [9, 17] [16, 24]. Mitophagy deficiency is known to lead to both mitochondrial dysfunction and defects in mitochondrial dynamics related to Miro1 [25, 26] [15]. We examined the removal of damaged mitochondria by mitophagy over time by measuring the protein levels of mitochondrial markers on the inner, outer membrane, matrix, and inter-membrane space. Knockdown of Miro1 in IKO mice significantly reduced the mitochondrial removal process, and all of the above mitochondrial markers disappeared in control islet cells faster than IKO islet cells. This indicates that mitophagy activity is weaker after Miro1 knockout. To further confirm this result, we monitored the autophagolysosome formation under an electric microscope. We found that knockout of Miro1 resulted in the accumulation of mitochondria that were damaged in the perinuclear region compared to control of islet cells, this is consistent with previous report that Miro1 is an essential for mitochondrial autophagy [27, 28]. Recent study shows that Miro1 involved as part of a mitophagy pathway by arresting of mitochondrial motility, helps to quarantine a depolarized mitochondrion or mitochondrial fragment prior to phagocytosis [29, 30], so that the damage caused by the released mtROS will be limited to a smaller area. Removal of Miro1 from beta-cell mitochondria will detaches kinesin from its surface [30]. This confirms our observations of defective insulin release, activated inflammation, and oxidative stress related to islet cell mitophagy function in IKO mice. This may be at the stage of the pathway at which decreased level of Miro1 occurs under more than 4-week sustained HFD stress and mitophagy impairs after initial compensation. Our findings underscore the significance of Miro1-associated mitophagy for providing adequate energy for a change in energy demand and clearing damaged mitochondria under late-event (4-24 week) of HFD stress.

Insulin function is synergistically coordinated by molecular events associated with IRS [31]. On the basis of previous studies and our current findings, JNK1 effectively promotes IRS1 phosphorylation of serine 307 after activation by Miro1 and subsequently induces inhibition of IRS1 tyrosine phosphorylation, which leads to compromised capacity of IRS1 to bind to insulin receptors and decreased AKT phosphorylation [32]. Akt phosphorylates Foxo1 and inhibits the transcriptional activity of Foxo1, which regulates various physiological functions such as energy metabolism. Akt → Foxo1 phosphorylation mediates many biological responses to insulin, and it serves as an indicator of insulin sensitivity [33]. Consistent with our results, it has been demonstrated that deletion of IRS1 and IRS2 genes in mice prevented activation of liver Akt-Foxo1 phosphorylation and led to the development of diabetes [34]. However, the molecular pathway of Miro1-regulated IRS-Akt-Foxo1 remains to be further elucidated [35].

In conclusion, this study determined a loss of Miro1-mediated effect on abnormal islet beta cell function *in vivo* which promotes unbalanced downstream MKK-JNK activation and IRS-AKT-Foxo1 inhibition, thereby promoting insulin resistance, mitophagy deficiency, and inflammatory response in the islet on an HFD stimulus. Thus, targeting Miro1 in the islet might be a promising therapeutic treatment for insulin resistance and related metabolic diseases.

## MATERIALS AND METHODS

### Mice

Generation of Miro1f/+ mice is described previously (18). The db/db and RIP-cre mice used in the present study were purchased from the biomedical institute of Nanjing University (stock no.: N000098, N000117). All experimental procedures were approved by Wuhan University Animal Care and Use Committee, and were conducted in full accordance with the Guide for the Care and Use of Laboratory Animals. All controls were phenotypic wild-type (WT) littermates of the mutant animals (Miro1F/+, RIPcre+/+). Male mice of 4-12 weeks of age (20-34 g) were used in these experiments. The mice were bred in a standard environment with a 12-h light/dark cycle. The diabetic model was established in mice through feeding a HFD (protein, 18.1%; fat, 61.6%; carbohydrates, 20.3%; D12492, Research Diets, NJ, USA) continuously for 24 weeks. Mice administered an NC diet (protein, 18.3%; fat, 10.2%; carbohydrates, 71.5%; D12450B, Research Diets) served as controls. Food and water were provided ad labium. The body weight (BW) and fasting blood glucose (FBG) level of mice was examined every 4 weeks. Immediately after death, the pancreases were surgically removed, and the islets isolated by collagenase dispersion. Krebs-Ringer bicarbonate buffer was used for isolation and pooling of the islets (19) .

### Cell culture and transfection

Islet cells or rat insulinoma (INS-1) (823/13) cells were grown in RPMI 1640 medium. For the different experiments, cells were cultured in 6-well plates until reaching 80% confluence before additional treatment. The samples were incubated for 6h or 12 h (37°C and 5% CO2) in the presence of 11mmol/l glucose, 0.05 mmol/l palmitate, or/and 10 μmol/l diphenylene iodonium (DPI), an NADPH oxidase inhibitor, Bay11-7082 (6.3μm) for 1h where indicated (20). The pmCherry-GFP-LC3B and pLVX-IRES-mCherry vectors used in the experiment were purchased from Addgene (Cambridge, MA, USA), and the construction of the infected plasmids pLVX-GFP-LC3B-IRES-mito-mCherry were described previously [36]. Lentiviral production was achieved through calcium phosphate transfection of four plasmids, according to the manufacturer’s instructions [37].

### Glucose and insulin tolerance tests

GTT and ITT performed upon intraperitoneal injection of 2 mg/g BW of D-glucose or 0.75 mU/g BW of insulin, respectively, as previously described (22). Tail vein blood was collected for measurements.

### Metabolic assays and serum cytokine analyses

FBG and fasting serum insulin (FINS) levels were examined after mice had fasted for 6 h by using a glucometer (Life Scan, PA, USA) and by ELISA (Millipore, MA, USA), respectively, and the insulin resistance index (HOMA-IR) was calculated with the equation (FBG (mmol l-1) x FINS(mIU l-1))/22.5. Blood glucose levels were examined at 0, 15, 30, 60, and 120 min after injection. ELISA (Invitrogen, CA, USA) examined the concentrations of cytokines (TNF-a, IL-1b, IL-6, and IL-10, and monocyte chemotactic protein- 1) in the serum. Commercial kits were employed to measure the TG in the islet (290-63701, Tokyo, Japan) according to the manufacturer’s instructions.

### H&E staining, immunostaining

For immunostaining, islets were handpicked under a stereomicroscope and fixed in a 10% formaldehyde solution. Islets were stained with primary antibodies against insulin followed by Alexa Fluor 488 secondary antibody staining and DAPI was used to visualize nuclei. Sections or islets were visualized with an AxioImager.

### Quantitative real time PCR (qRT-PCR)

Total RNA was extracted using Trizol Reagent (Invitrogen, USA) and cDNA was synthesized using iScript™ cDNA Synthesis Kit (Bio-Rad, USA) according to the manufacturer’s instructions. mRNA expression was normalized to that of 18S mRNA.

### Confocal microscopy

Fixed and permeabilized islets cells were used for confocal microscopy as described previously with modification (23). The fixed cells were blocked with normal goat serum, probed with mouse anti-Miro1 or insulin monoclonal antibodies and stained with Texas red-conjugated goat anti-mouse IgG antibodies (KPL, Gaithersburg MD). Finally, the cover glass was washed, mounted and examined using the confocal laser microscope.

### Detection of reactive oxygen species

Treated islets were washed in PBS and placed in PBS containing 2.5 μM MitoSOX (to measure mitochondrial ROS production) and/or 10µm 6-carboxy-2’, 7’-dichlorodihydrofluorescein diacetate, di(acetoxymethyl ester) (DCF-DA, Invitrogen, Carlsbad, CA) (to measure total cellular ROS) for 30 min at 37°C. Following a PBS wash and re-incubation in PBS for 1h at 37°C, 10µM Hydrogen Peroxide (H_2_O_2_) was used for 2 h as a positive control. Fluorescence was measured on a SpectraMax M5 plate reader (Molecular Devices, Sunnyvale, CA); emission wavelength 480nm; excitation wavelength 530nm.

### Western blotting

Cell lysates were separated by electrophoresis prior to transfer to PVDF membranes. Data were normalized relative to actin or GAPDH. The following primary antibodies were used for Western blots and immunostaining: anti-LC3 (Cell Signaling, 4108), anti-Tom20 (SantaCruz, sc-17764); anti-calregulin (SantaCruz, sc-6468-R); anti-Gapdh (Abcam, ab9483); anti-Golgi58 (Abcam, ab27043); rabbit anti-Miro1 (Sigma, HPA01068); p-JNK (Cell Signaling, 4668); JNK (Cell Signaling, 9252); p-IRS1ser307 (Cell Signaling, 2831); IRS1 (Cell Signaling, 2832); ERK1/2, p38, MEK1/2 antibodies ( New England Biolabs, p44/42, 9102L, 4694S), p-AKTSer473 (Cell Signaling, 4060); AKT (Cell Signaling, 4691); Antibody against p-IRS1Tyr608 (Cell Signaling, 09-432); anti-p-POXO1 (ORIGENE, TA323072). Anti-IL1 beta antibody (Abcam, ab2105); anti-IL6 antibody (Abcam, ab6672); Anti-monocyte chemotactic protein-1(MCP)-1 antibody (Abcam, ab9669); Anti-TNF alpha antibody (Abcam, ab9635). Alexa Fluor-conjugated antibodies (Molecular Probes, A21057, A21076, A21096) were used as secondary antibodies.

### Mitochondrial isolation

Mitochondria were isolated using mitochondrial isolation kit according to the manufacturer’s instructions (Pierce). Briefly, cells were homogenized and then centrifuged at 750 x g for 10min at 4°C. The supernatant was further centrifuged at 12,000 x g for 15min at 4°C. The pellet was then washed and kept as the mitochondrial fraction. The supernatant was further centrifuged at 100,000 x g for 1h at 4°C and designated as the cytosolic fraction.

### Electron microscopy

Islets from IKO and control mice were cultured in Aclar embedding film (2-mm thickness, Electron Microscopy Sciences, PA, USA), fixed in 2.5% glutaraldehyde and 4% sucrose in a 0.05 mol/l phosphate buffer, pH 7.4, and examined with a JEOL 1200EX electron microscope (JEOL, Tokyo, Japan) as described previously.

### Flow cytometry

MitoSOX, MitoTracker green and red, and DCFDA staining were done according to manufacturer’s instructions (Invitrogen). Data were acquired with a FACS Calibur flow cytometer (BD Biosciences).

### Statistical analysis

All values are expressed as mean ± SD. Differences between means were analyzed using either one-way or two-way ANOVA followed by Newman-Keuls post-hoc testing for pair-wise comparison using SPSS. The null hypothesis was rejected when the *P*-value < 0.05.

## SUPPLEMENTARY MATERIALS FIGURES



## Abbreviations

T2D: type 2 diabetes
Miro1: mitochondrial rho GTPase 1
NLRP3: NOD-like receptor 3
IKO: Miro1 ablation in islets
HFD: high fat and diet
mtROS: mitochondrial ROS
INS: rat insulinoma cell
PA: palmitic acid
HFG: high fat and glucose
