# Supplementary material for: Inhibition of Miro1 disturbs mitophagy and pancreatic β-cell function interfering insulin release via IRS-Akt-Foxo1 in diabetes
Source: Oncotarget. 2017 Sep 16;8(53):90693–705. doi: 10.18632/oncotarget.20963 (PMC5710878; doi:10.18632/oncotarget.20963)
Supplement: Supplementary file 1 [file oncotarget-08-90693-s001.pdf]

Inhibition of Miro1 disturbs mitophagy and pancreatic β-cell function interfering insulin release via IRS-Akt-Foxo1 in diabetes

SUPPLEMENTARY MATERIALS

S1. PA-BSAsolutionpreparation

Sodium PA was dissolved in 95% ethanol at 60°C to yield a stock concentration of 10mM and kept at 20°C. Nitrogen gas was used to completely remove ethanol from PA stock solution. PA was then conjugated with fatty acid-free BSA at a 3:1 molarratio.

S2. H&E staining and immunostaining staining

Pancreas samples from mice were embedded in OCT medium (TissueTek) and snap frozen in liquid nitrogen. Samples were sectioned while frozen into 8 μm slices in a cryotome and fixed with a 10% formaldehyde solution. H&E staining of pancreas were performed as described previously. Pictures were taken with a Hamamatsu Orca camera. Average islet size and frequency were calculated by NIH ImageJ software after measuring and counting 100 islets per pancreas cryosection.

For immunostaining, islets were handpicked under a stereomicroscope and fixed in a 10% formaldehyde solution. Islets were stained with primary antibodies against insulin (Cell Signaling) followed by Alexa Fluor 488 secondary antibody staining or glucagon (Abcam) followed by Alexa Fluor 495 secondary antibody and DAPI was used to visualize nuclei. Sections or islets were visualized with an AxioImager.

S3. A mouse model of STZ-induced diabetes

The diabetic mice were developed by intraperitoneal injection of freshly prepared STZ (Sigma-Aldrich, USA) dissolved in a citrate buffer (0.1 M, pH4.5) at a dose of 40 mg/kg body weight after an overnight fast for 5 days [22]. STZ-injected animals were administration oral 2.5 g/kg glucose solution 4 hours later to prevent initial drug-induced hypoglycemic mortality. The blood glucose level was monitored 72 h after the STZ injection using a glucometer and mice with a fasting glucose level greater than 11.1 mmol/L were used as the diabetic mice for further experiments. Three days after the STZ injection, agent treatment was performed.

S4 Primes for QPCR:

Mdh2
F: TGAGGTTCCCATCACCTCTC
R: CTGTGGGGTTTGCTTTTGTT
Sdhb
F: CAACGTGCAACAAAGATGCT
R: GGGGGTGACTGACTCTGTGT
Vamp2
F: GAACAAAGTTCGCTCCAAGC
R: ATTCCTCACTGGTCGTGGTC
GAPDH
F: ACTCCACTCACGGCAAATTC
R: TCTCCATGGTGGTGAAGACA
SNAP25
F: AGATGCCTTTGAAACCGATG

R: TTGGGCCTCTCTAATACCCA
Stx1a
F: GCTGCTTGCACATTTGTGTT
R: TGAGTGACTGGTGGGAAGAA
Acadvl
F: CCAATGCCGTTCTCAAAATC
R: ATGCACTACCAGGACAGCCT
Cpt2
F: TATCAGAGAAGCCAGCTCCC
R: TAGAGCTGCACTTTCGGGTT
PDX-1
F: AGCAGTACTACGCGGCCACA
R: GCACTTCGTATGGGGAGATG
18S RNA
F: AAACGGCTACCACATCCAAG
R:CCTCCAATGGATCCTCGTTA
INS-1
F:CTTGCCCTCTGGGAGCCCA
R:TGAAGGTCCCCGGGGCTTC
Glut2
F: TCAGAAGACAAGATCACCGG
R:GTGAGCAGATCCTTCAGTCT
Cx36:
F: GACCATCTTGGAGAGGCTGC
R:ACCACCACAGTCAACAGGATCC
NeuroD:
F: ACGCAGAAGGCAAGGTGTC
R-CCGCTCTCGCTGTATGATTT
Urocotin-3
F: GTTTCACATCCTTCTTTTCTAC
R:CTTGTCTTGATGTGCCACCCTC

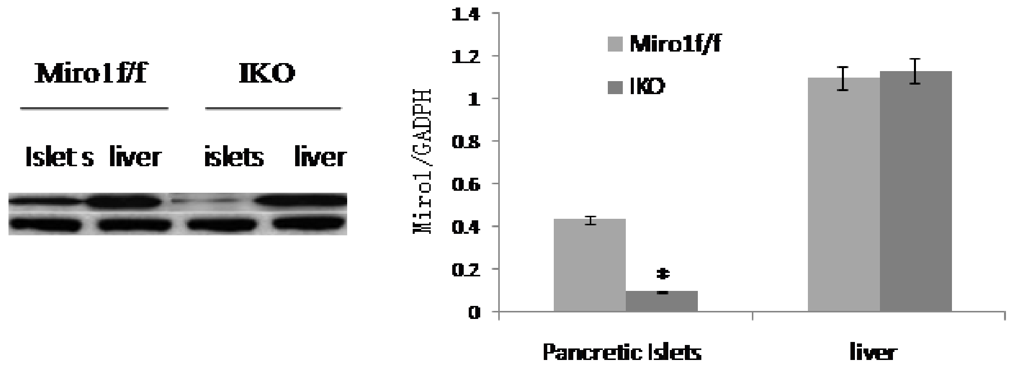

Supplementary Figure 1: Miro1 expression in the islet and liver samples of Miro1f/f and IKO mice were examined by western blot analysis (n=4). *P<0.05 vs. Miro1f/f grou

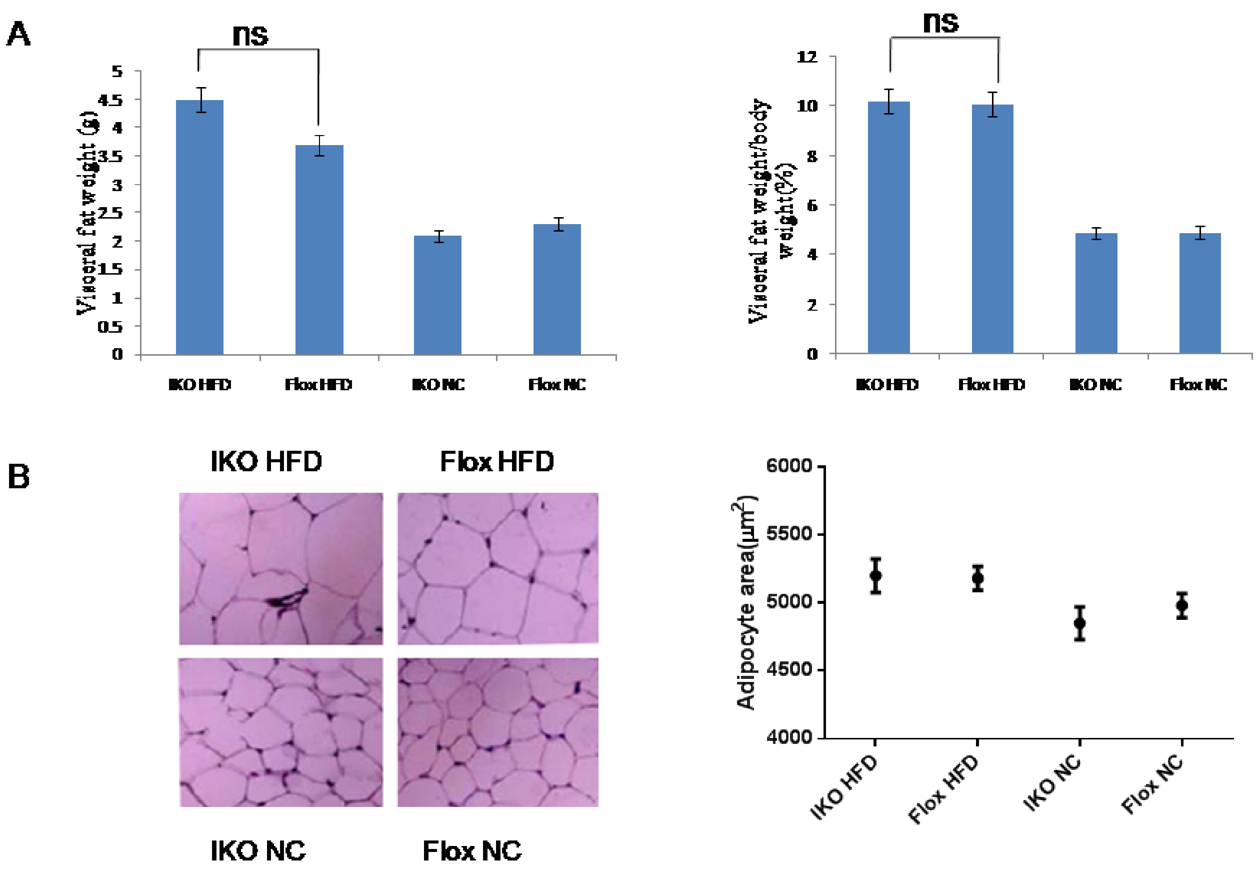

Supplementary Figure 2: IKO promotes weight gain of adipose tissue without changing adipocytes enlargement under HFD. (A) Visceral fat weights of IKO, Flox, and their littermate controls after a 24-week of HFD or NC treatment. n=11-23/pre group. (B) The representative H&E staining images (upper panel) and the analyzed adipocyte areas (bottom column plots) on the white adipose tissue (WAT) sections of mice in the indicated groups. n=5-9for each group. # P<0.05 vs. Flox/HFD group. n.s., no significant difference. The data represent as the mean ± SD Significance determined by two-way analysis of variance with general linear model procedures using a univariate approach.
